# Preventive health screenings in early detection of type 2 diabetes: A prospective study

**DOI:** 10.6026/973206300212469

**Published:** 2025-08-31

**Authors:** Akhshayaa Gunasekaran, Deepika Muthu, Shaheed Shaik, Gopikrishnan Sumej Babu, Mohammed Zakiullah Shareef, Hari Laxman Kanthavel, Tanvi Ghanshyambhai Patel

**Affiliations:** 1 Department of Paediatrics, Prince Charles Hospital, Cwm Taf Morgannwg University Health Board, Wales, UK; 2 Department of Critical Care Medicine, Aster Whitefield Hospital, Bangalore, Karnataka, India; 3 Department of General Medicine, Gandhi Medical College, Secunderabad, Telangana, India; 4 Department of General Medicine, Swansea Bay University Health Board, Wales, United Kingdom; 5 Department of Internal Medicine, AL Hada Armed Forces Hospital, TAIF, Saudi Arabia; 6 Department of General Medicine, Sir Ivan Stedeford Hospital, Ambattur, Chennai, India; 7 Department of Medicine, ITM Medical Hospital, Vadodara, Gujarat, India

**Keywords:** Preventive screening, type 2 diabetes, early detection, HbA1c, public health

## Abstract

The effectiveness of routine preventive health screenings in identifying undiagnosed type 2 diabetes among 135 adults over 24 months is reported. Participants underwent annual fasting glucose, HbA1c, and BMI assessments. Early-stage type 2 diabetes was detected in 23.7% of cases through screening alone. Significant correlations were found between undiagnosed diabetes and elevated BMI and family history. Thus, we show the value of preventive screening in early intervention and diabetes control.

## Background:

Type 2 diabetes mellitus (T2DM) is a chronic metabolic disorder characterized by insulin resistance and progressive pancreatic β-cell dysfunction, leading to hyperglycemia and associated complications [1]. It has become a significant public health concern worldwide, with rising incidence due to urbanization, sedentary lifestyles and dietary changes [2]. A major challenge in managing T2DM is the substantial proportion of individuals who remain undiagnosed until complications arise [3]. Early detection of T2DM is crucial for initiating timely lifestyle modifications and pharmacological interventions that can delay disease progression and prevent complications such as cardiovascular disease, nephropathy, neuropathy and retinopathy [4]. Preventive health screenings-especially that involving fasting plasma glucose (FPG), glycated hemoglobin (HbA1c) and body mass index (BMI)-offer a practical approach for identifying individuals at risk or in the early stages of the disease [5]. Despite the availability of screening guidelines from health authorities such as the ADA and WHO, routine implementation remains inconsistent in many primary care settings [6]. Moreover, limited prospective data exist evaluating the real-world impact of preventive screenings on early diabetes detection rates [7]. Therefore, it is of interest to assess the utility of structured annual preventive screenings in detecting previously undiagnosed T2DM among adults, and to evaluate the association between screening findings and common risk factors such as age, BMI, physical activity level, and family history of diabetes.

## Materials and Methods:

This prospective observational study was conducted over a 24-month period at a tertiary care hospital and its affiliated community clinics. A total of 135 adults aged 30 to 60 years without a prior diagnosis of diabetes were recruited through outpatient screening. Participants underwent baseline evaluation followed by annual follow-ups that included fasting plasma glucose, HbA1c, BMI, blood pressure, and lifestyle assessments. Data on dietary patterns, physical activity, and family history of diabetes were collected through a structured questionnaire. Based on American Diabetes Association (ADA) criteria, individuals were categorized as normoglycemic, prediabetic, or diabetic. Those identified as diabetic through screening were referred for further evaluation and management. Statistical analysis was done using SPSS version 25, with significance set at p < 0.05.

## Results:

Out of 135 participants enrolled, 128 completed the 24-month follow-up. The mean age was 45.3 ± 8.1 years, and 58% were female. At baseline, all participants were non-diabetic by history. By the end of the study, 32 individuals (25%) were newly diagnosed with type 2 diabetes, and 41 (32%) with prediabetes. Significant associations were found between elevated BMI, sedentary lifestyle, and family history of diabetes with new-onset diabetes. Preventive screening facilitated early diagnosis in asymptomatic individuals.

Table 1 (see PDF) presents the glycemic status of participants at 24 months. It shows that 43 percent remained normoglycemic, 32 percent were classified as prediabetic, and 25 percent had progressed to a new diagnosis of type 2 diabetes, highlighting the distribution of long term glycemic outcomes in this cohort. Table 2 (see PDF) reports the association between BMI categories and diabetes diagnosis. Participants with obesity (BMI ≥ 30 kg/m^2^) exhibited the highest incidence of new T2DM (17/29), whereas those with normal BMI showed the lowest (5/43), underscoring the strong link between elevated body mass and diabetes risk. Table 3 (see PDF) illustrates the relationship between physical activity level and diabetes onset. Sedentary individuals experienced the greatest incidence of new cases (17/42) compared to moderately active (9/35) and active (6/51) groups, suggesting a protective effect of higher activity. Table 4 (see PDF) indicates that a positive family history markedly increases diabetes risk 22 of 51 participants with diabetic relatives developed T2DM versus only 10 of 77 without such history emphasizing genetic predisposition. Table 5 (see PDF) demonstrates mean HbA1c values at final follow up, which rose progressively from 5.4 percent in the normoglycemic group to 5.9 percent in prediabetes and 7.2 percent in newly diagnosed T2DM (p < 0.001), reflecting worsening glycemic control. Table 6 (see PDF) details mean fasting plasma glucose levels, which increased from 88.6 mg/dL (normoglycemic) to 105.3 mg/dL (prediabetic) and 138.5 mg/dL (new T2DM) (p < 0.001), mirroring the HbA1c trends. Table 7 (see PDF) examines diabetes diagnosis by gender and reveals a slightly higher incidence in males (18/57) than females (14/71) (p = 0.038), suggesting sex related differences in disease onset. Table 8 (see PDF) describes the age wise distribution of new T2DM diagnoses, which rose from 6 of 43 in the 30-39 year group to 12 of 46 in 40-49 years and 14 of 39 in 50-60 years, indicating increasing risk with age. Table 9 (see PDF) identifies obesity (OR 3.8) and positive family history (OR 2.9) as independent predictors of new T2DM in multivariate logistic regression analysis, underscoring the multifactorial nature of disease risk. Table 10 (see PDF) explores symptom status at diagnosis among new T2DM cases and reveals that 84.4 percent were asymptomatic at detection, highlighting the importance of proactive screening.

## Discussion:

This prospective study highlights the significant role of preventive health screenings in the early detection of type 2 diabetes among adults without a prior diagnosis. Over the 24-month period, a notable 25% of participants were newly diagnosed with T2DM, and an additional 32% were identified as prediabetic. These findings support the hypothesis that structured annual screenings can uncover a substantial burden of undiagnosed glycemic abnormalities, particularly in individuals with high-risk profiles. The results reinforce the strong association between elevated BMI, sedentary lifestyle, and a positive family history with new-onset diabetes [8]. Participants classified as obese (BMI ≥30) had nearly four times the odds of being diagnosed with T2DM, aligning with existing literature that highlights obesity as a primary modifiable risk factor [9]. Similarly, sedentary individuals and those with a family history of diabetes demonstrated significantly higher rates of disease detection, emphasizing the relevance of targeted screening in these subgroups [10]. Importantly, the majority of newly diagnosed participants were asymptomatic at the time of detection, underscoring the silent progression of early diabetes and the inadequacy of symptom-based diagnosis [11]. These results suggest that without routine screening, these cases would likely have gone undetected until complications developed [12]. The mean HbA1c and fasting glucose levels in diagnosed individuals further confirm their biochemical disease state, validating the effectiveness of the screening tools employed [13]. Our findings are consistent with global recommendations advocating for opportunistic diabetes screening in primary care settings, especially in populations with known risk factors. Despite these recommendations, implementation often remains inconsistent due to logistical and economic barriers. This study demonstrates that preventive screenings can be successfully integrated into routine healthcare visits and can yield high diagnostic yield at relatively low cost.

## Conclusion:

Preventive health screenings are effective in identifying undiagnosed type 2 diabetes and prediabetes, especially among high-risk individuals. Early detection through routine testing enables timely intervention, reducing the risk of future complications. Thus, integrating structured screenings into primary healthcare can play a vital role in improving public health outcomes.
